# Muscle wasting in myotonic dystrophies: a model of premature aging

**DOI:** 10.3389/fnagi.2015.00125

**Published:** 2015-07-09

**Authors:** Alba Judith Mateos-Aierdi, Maria Goicoechea, Ana Aiastui, Roberto Fernández-Torrón, Mikel Garcia-Puga, Ander Matheu, Adolfo López de Munain

**Affiliations:** ^1^Neuroscience Area, Biodonostia Health Research InstituteSan Sebastián, Spain; ^2^CIBERNED, Instituto Carlos III, Ministerio de Economía y CompetitividadMadrid, Spain; ^3^Cell Culture Platform, Biodonostia Health Research Institute, San SebastiánSpain; ^4^Department of Neurology, Hospital Universitario Donostia, San SebastiánSpain; ^5^Oncology Area, Biodonostia Health Research InstituteSan Sebastián, Spain; ^6^Department of Neuroscience, Universidad del País Vasco UPV-EHUSan Sebastián, Spain

**Keywords:** myotonic dystrophy, aging, muscle wasting, satellite cells, sarcopenia

## Abstract

Myotonic dystrophy type 1 (DM1 or Steinert’s disease) and type 2 (DM2) are multisystem disorders of genetic origin. Progressive muscular weakness, atrophy and myotonia are the most prominent neuromuscular features of these diseases, while other clinical manifestations such as cardiomyopathy, insulin resistance and cataracts are also common. From a clinical perspective, most DM symptoms are interpreted as a result of an accelerated aging (cataracts, muscular weakness and atrophy, cognitive decline, metabolic dysfunction, etc.), including an increased risk of developing tumors. From this point of view, DM1 could be described as a progeroid syndrome since a notable age-dependent dysfunction of all systems occurs. The underlying molecular disorder in DM1 consists of the existence of a pathological (CTG) triplet expansion in the 3′ untranslated region (UTR) of the *Dystrophia Myotonica Protein Kinase* (*DMPK)* gene, whereas (CCTG)n repeats in the first intron of the* Cellular Nucleic acid Binding Protein/Zinc Finger Protein 9* *(CNBP/ZNF9)* gene cause DM2. The expansions are transcribed into (CUG)n and (CCUG)n-containing RNA, respectively, which form secondary structures and sequester RNA-binding proteins, such as the splicing factor muscleblind-like protein (MBNL), forming nuclear aggregates known as foci. Other splicing factors, such as CUGBP, are also disrupted, leading to a spliceopathy of a large number of downstream genes linked to the clinical features of these diseases. Skeletal muscle regeneration relies on muscle progenitor cells, known as satellite cells, which are activated after muscle damage, and which proliferate and differentiate to muscle cells, thus regenerating the damaged tissue. Satellite cell dysfunction seems to be a common feature of both age-dependent muscle degeneration (sarcopenia) and muscle wasting in DM and other muscle degenerative diseases. This review aims to describe the cellular, molecular and macrostructural processes involved in the muscular degeneration seen in DM patients, highlighting the similarities found with muscle aging.

## Introduction

Myotonic dystrophy type 1 (DM1), also known as Steinert’s disease (OMIM: 160900), is a dominantly inherited multisystem disease. DM1 is the most common form of adult-onset muscular dystrophy; it affects one out of 8000 people worldwide, with an even greater prevalence in some specific areas such as Quebec (Canada) and the Basque Country (Spain) (López de Munain et al., 1993; Mathieu and Prévost, 2012). The DM1 phenotype shows an extremely wide variability among affected patients, with some being asymptomatic while others have severe congenital forms. Patients are classified into four categories regarding the age of onset of symptoms: late-onset, adult-onset, childhood-onset and congenital forms (Harper, 2001; Arsenault et al., 2006).

**Figure 1:**
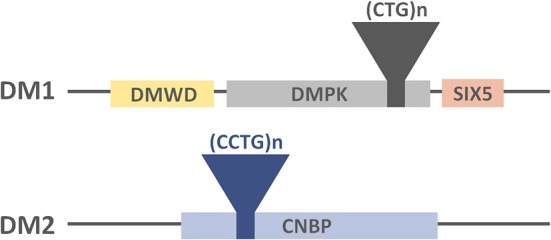
**Representation of the DM-causing genes, the location of the tandem repeats and their neighboring genes**.

The disease is caused by an unstable expansion of a trinucleotide (CTG) repeat motif located in the 3′ untranslated region (UTR) of the *Dystrophia Myotonica Protein Kinase (DMPK)* gene (Brook et al., 1992; Figure 1). Unaffected individuals carry less than 50 triplet repeats, whereas expansions ranging between 50 and 4000 CTG repeats have been found in affected individuals. Importantly, the length of CTG expansion is associated with the age of onset of the disease and its severity (Martorell et al., 1998).

DM1 patients experience a progressive dysfunction of multiple organs and tissues, including skeletal, cardiac and smooth muscles, the endocrine system, eyes, gonads, the central nervous system (CNS), and an increased risk of developing neoplasias (López de Munain et al., 1993; Harper et al., 2002; Gadalla et al., 2013). Thus, DM1 somewhat resembles a progeroid syndrome, defined as an accelerated aging and dysfunction of several systems.

A second form of myotonic dystrophy exists, DM type 2 (OMIM: 602668), initially named proximal myotonic myopathy due to the greater weakness of proximal as compared to distal muscles (Ricker et al., 1994). DM2 patients also develop a multisystem dysfunction but they generally experience a milder phenotype as compared to DM1 (Table 1). Consistent with this, congenital and childhood-onset forms of DM2 are absent, and the disease phenotype ranges from early adult-onset severe forms to very late-onset mild forms (Day et al., 2003). The prevalence of this disorder has yet to be clearly defined but it is estimated to be similar to DM1 in Northern European Countries (Udd and Krahe, 2012). However, very late-onset forms of this disease might often go undiagnosed due to its mild phenotype, which can be concealed by other age-related dysfunctions. Unlike what occurs in DM1, aging-like symptoms in DM2 might not be so evident. Regarding the muscle-specific phenotype, it is unknown why distal muscles are predominantly affected in DM1 patients whereas DM2 patients show a more proximal affectation.

**Table 1:** **Summary of main clinical features that differ between both DM forms**.

Features	DM1	DM2
Age of onset	At any age	Adulthood
Congenital forms	Yes	No
Gene expansion	DMPK, (CTG)n	CNBP, (CCTG)n
Predominantly affected muscles	Distal	Proximal
Predominantly affected fibers	Type 1	Type 2

As is the case with the genetic origin of DM1, DM2 is caused by a tetranucleotide (CCTG) expansion in intron 1 of the *Nucleic Acid-binding Protein (CNBP or ZNF9)* gene (Liquori et al., 2001; Figure 1). Healthy individuals carry less than 30 tetranucleotide repeats, whereas repetition lengths between 55 and 11000 have been found in affected patients (Liquori et al., 2001).

Skeletal muscle, one of the most severely affected tissues in these diseases, may age prematurely in DM patients, mimicking sarcopenia, known as the age-related loss of muscle mass and function (Evans and Campbell, 1993; Fielding et al., 2011). This syndrome can refer to loss of muscle mass alone or in conjunction with fatty substitution (Fielding et al., 2011). Although the causes of sarcopenia have not as yet been accurately determined, several age-related factors, such as muscle disuse, nutritional deficiencies, hormonal changes and insulin resistance could notably contribute to its onset.

## Multisystem Dysfunction in DM

**Figure 2:**
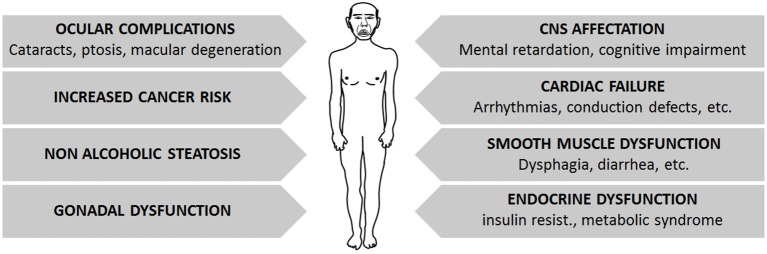
**Summary of main symptoms affecting DM patients, which constitute the multisystem affectation found on them**.

The previously described genomic tandem repeats lead to the progressive degeneration of several tissues and organs, which is more prominent in DM1 and milder in DM2 patients (Figure 2).

DM patients may suffer a broad variety of symptoms affecting the three muscle types: cardiac, skeletal and smooth muscles. Cardiac failure is common in DM1 patients, often manifested as arrhythmias and conduction defects (Antonini et al., 2000; Mammarella et al., 2000; Pelargonio et al., 2002; Groh et al., 2008; Cudia et al., 2009; Petri et al., 2012). Although congestive heart failure is a rare complication, subclinical systolic dysfunction as shown by echocardiographic or magnetic resonance imaging is frequent (De Ambroggi et al., 1995; Tokgozoglu et al., 1995; Bhakta et al., 2004; Hermans et al., 2011; Petri et al., 2012). The frequency of heart failure correlates with age, male gender, length of the tandem repeat sequence and the degree of neuromuscular disability (Tokgozoglu et al., 1995; Antonini et al., 2000; Groh et al., 2008; Cudia et al., 2009; Kaminsky and Pruna, 2012). Symptoms involving the smooth muscle, such as dysphagia, constipation, intestinal pseudo-obstruction and diarrhea, are also relatively frequent in DM1 patients (Bujanda et al., 1997; Bellini et al., 2006; Ercolin et al., 2013). As for skeletal muscle involvement, both DM forms share common muscle histopathologic features, with a markedly increased variation of fiber diameter and prominent central nucleation, which is a feature of constantly regenerating muscles with immature fibers. Other signs, such as the presence of basophilic regenerating fibers, branched fibers, and adipose and fibrotic tissue can also be found to varying degrees, depending on the extent of muscle degeneration and the severity of the disease. Although DM1 affects mainly distal muscles and DM2 affects proximal muscles, only a few DM type-specific features have been described. Among these, ring fibers (peripheral myofibers that surround other fibers) and sarcoplasmic masses are more frequently seen in DM1 muscles. Nuclear clumps (condensed chromatin structures in the myonuclei, indicating cell death) are found in DM2 patients even when muscular weakness is not clinically evident, whereas they arise later in DM1 patients, mainly in end-stage muscles (Vihola et al., 2010). Moreover, it must be noted that DM1 muscles show a prominent loss of type 1 fibers, whereas type 2 fibers are predominantly affected in DM2 patients (Vihola et al., 2003; Schoser et al., 2004; Bassez et al., 2008; Pisani et al., 2008). Interestingly, the histology of DM muscles resembles that of aged muscles, with fiber size variability, centrally located nuclei with chromatin clumps and fiber atrophy. Muscle regeneration also seems to be decreased in both conditions, probably due to satellite cell dysfunction, which may fail to activate and/or differentiate to muscle upon myogenic stimuli (Huichalaf et al., 2010; Malatesta, 2012; Malatesta et al., 2014).

As part of the multisystem involvement, many DM1 patients show insulin resistance due to the aberrant splicing of the insulin receptor (IR) mRNA, which is highly expressed in skeletal muscle. Consequently, patients show a reduced responsiveness to insulin as compared to healthy individuals (Morrone et al., 1997; Savkur et al., 2001).

The CNS is also negatively affected in DM1 patients. The large majority of congenital and childhood-onset DM1 patients suffer mental retardation, whereas patients with the adult-onset forms may show varying degrees of cognitive dysfunction, where a positive correlation is observed between triplet expansion length and patients’ age. Cognitive dysfunction is characterized by a dysexecutive syndrome with predominant frontoparietal involvement (Sistiaga et al., 2010). Moreover, DM1 patients go through behavioral-personality changes (e.g., reduced initiative, inactivity, apathic temperament and paranoid personality traits) and excessive daytime sleepiness. There is tentative data supporting an age-dependent decline of cognitive functioning in DM1 patients (Modoni et al., 2008), possibly associated with the degeneration of the diffuse (predominantly temporo-insular) subcortical white matter, and a reduction of the cerebral blood flow in frontal areas (Romeo et al., 2010).

DM1 patients also present hepatic involvement. Indeed, 66% of patients show abnormal hepatic enzyme levels and non-alcoholic steatosis (Achiron et al., 1998). Ocular complications, including ptosis, weakness of the ocular muscle and cataracts are also common in DM1 patients, and other less frequent features, such as retinal changes or macular degeneration, may also be present in these patients (Kimizuka et al., 1993; Krishnan and Lochhead, 2010).

Finally, DM patients may also suffer fertility dysfunction. Approximately two thirds of affected males have reduced sperm quality as a result of testicular atrophy (Pan et al., 2002). Affected female fertility is less well documented (Verpoest et al., 2008), but the length of triplet expansion does not seem to be correlated with this aspect of the disease. Importantly, the age of the pregnant patient and parity significantly affect the live birth delivery rate (Verpoest et al., 2010).

In order to decipher why some specific tissues are more severely affected than others in these diseases, it must be highlighted that some tissues and cell types possess a higher tendency to extend these tandem repeat sequences. This leads to the existence of cells with different repeat lengths within an organism, known as *somatic mosaicism*. The longest tandem repeats have been found in severely affected tissues; indeed, skeletal muscle cells possess considerably longer repeat sequences as compared to other cell types (Anvret et al., 1993; Thornton et al., 1994). Importantly, cells with longer tandem repeats tend to accumulate more repetitions than cells with shorter repeat sequences (Monckton et al., 1995), thus aggravating the degenerative state of predominantly affected tissues in these patients, such as the CNS and cardiac and skeletal tissues.

In addition to somatic cells, germline cells are also prone to genomic instability and thus accumulate tandem repeats in DM. This leads to *anticipation*, which refers to the increase in disease severity and decrease in the age of onset in each generation of affected families (López de Munain et al., 1994).

## Repeat Expansion Mechanisms in DM

As previously mentioned, somatic mosaicism is an important feature of myotonic dystrophies; tandem repeats increase with aging in an unsynchronized fashion, leading to cells with different repeat lengths in their genomes. Furthermore, somatic instability is prevalent in highly affected tissues, such as skeletal muscle (Morales et al., 2012).

Slippage of DNA polymerase during DNA replication was at first considered the main mechanism through which repeat sequences are expanded in myotonic dystrophies. Therefore, highly proliferative cells would have a higher tendency to expand DNA repeat sequences, which contradicts the fact that the longest tandem repeats are found in severely affected tissues that happen to be post-mytotic. In this regard, DNA repair mechanisms, which are also active in non-cycling cells, have been found to notably contribute to this phenomenon (van den Broek et al., 2002; Savouret et al., 2003; Kovtun et al., 2004; Seriola et al., 2011; Gomes-Pereira et al., 2014), which could explain the elongation of tandem repeats in non-proliferating cells in culture (Gomes-Pereira et al., 2014).

DNA tandem repeats in DM acquire secondary conformations, usually forming hairpin structures. DNA repair proteins recognize these structures and may abnormally repair them, varying the repeat length (McMurray, 2008). Knock-out animal models for components of the DNA repair machineries, as well as silencing these genes in patient-derived cultures, reduce or even abrogate the elongation of repeat expansion sequences. For example, it has been established that Msh2, a component of the mismatch repair (MMR) machinery, is required for triplet expansion in DM1-derived pluripotent stem cells (iPS) cells, as its silencing blocks the expansion of the triplet repeat sequence (Du et al., 2013). Knock-down and overexpression experiments of proteins involved in Base Excision Repair (BER) systems in yeast also indicate that these proteins might play a role in the expansion of repeat sequences (Refsland and Livingston, 2005; Subramanian et al., 2005). However, this fact has not been fully demonstrated in mice due to the lethality of knock-out mouse models for these proteins (Xanthoudakis et al., 1996; Tebbs et al., 1999).

The fact that skeletal muscle is composed of postmitotic cells suggests that these DNA repair systems may contribute in a major way to the expansion of repeat sequences in these tissues. Interestingly, a study performed by Vahidi Ferdousi et al. (2014) shows that double-strand breaks produced by irradiation are more rapidly repaired in muscle satellite cells than their progeny, thus indicating that satellite cells possess a more efficient DNA repair system, which usually acts through non-homologous end joining. Therefore, it could be hypothesized that triplet expansions are predominantly elongated in satellite cells due to the high activity of the DNA-repair system in these cells. This is consistent with the muscle degeneration seen in DM patients, as dysfunction of satellite cells would disrupt muscle regeneration.

Recent studies have shown that, in addition to DNA repair molecules, the 26S proteasome also participates in the expansion of tandem repeat sequences (Debacker et al., 2012; Concannon and Lahue, 2013). Importantly, the proteolytic activity of 26S, rather than the stress-induced activation of the ubiquitin-proteasome system (UPS), is implicated in this elongation process. Indeed, DNA repair mechanisms could interact with the proteasome to induce repeat expansions through Rad23, a protein involved in both nucleotide excision repair (NER) system and the carriage of ubiquitinated proteins to the proteasome (Concannon and Lahue, 2014).

## Pathogenic Mechanisms in DM

It is remarkable that severely affected tissues in DM possess very low mitotic rates. Interestingly, *in vitro* experiments have shown that nuclear foci tend to predominantly accumulate in non-cycling cells. Cycling DM2 fibroblasts accumulate nuclear foci during interphase but these are cleared out during mitosis, leading to mitosis-dependent cycles of foci formation and disassembly. On the contrary, mitotically arrested fibroblasts accumulate more foci, which keep enlarging progressively (Giagnacovo et al., 2012). In this regard, muscle biopsies of DM2 patients have confirmed that the size of myonuclear foci increases in an age-dependent manner (Giagnacovo et al., 2012). Therefore, this could help clarify why tissues and cell types with low mitotic rates are preferentially affected in these diseases.

Several mouse models have been created to study the pathogenic mechanisms of DMs. These models can be categorized into 4 subgroups regarding the genetic modifications that have been introduced in order to mimic different molecular aspects of the diseases: (i) introduction of unstable CTG repeat sequences; (ii) overexpression of toxic CTG/CCTG repeats; (iii) inactivation of the genes located in the DM1 locus; and (iv) mice models of MBNL inactivation or CUGBP1/CELF overexpression (Gomes-Pereira et al., 2011).

**Figure 3:**
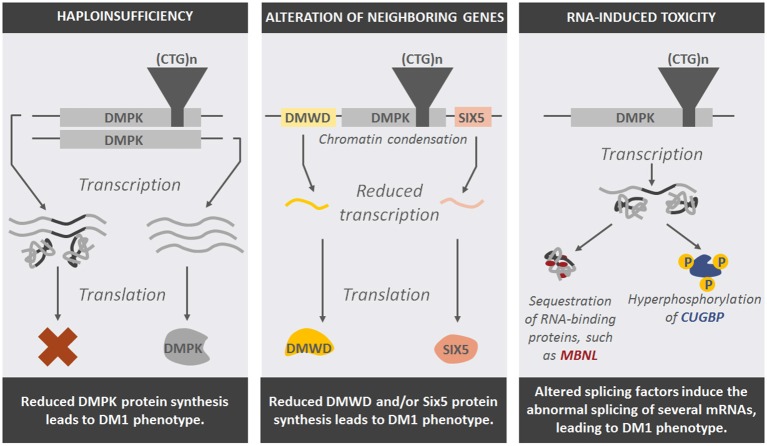
**Representation of potential pathogenic mechanisms that explain the effect of DNA expansions in DM1-affected cells and the phenotype seen in patients**.

Based on these studies, three pathogenic mechanisms that link the nucleotide expansion with the clinical manifestation of myotonic dystrophies have been proposed: (a) DMPK and CNBP haploinsufficiency; (b) *Cis* alteration of neighboring genes; and (c) RNA-induced toxicity (Figure 3).

### Haploinsufficiency

Haploinsufficiency refers to the deleterious effect of having only one wild type copy of a gene in the phenotype of the organism. DM patients carry only one wild type copy of the *MDPK* or *CNBP* gene, as the other copy harbors the tandem repeats that form nuclear foci, therefore reducing the amount of DMPK or CNBP protein synthesized.

DMPKs is a serine-threonine kinase, whose function in skeletal muscle is not fully understood. It has been shown to localize in the nuclear membrane, where it interacts with lamin-A/C and its deficiency leads to nuclear envelope instability (Harmon et al., 2011). However, it has also been found in the cytoplasm and the cellular membrane during cell division and cardiomyocyte differentiation (Harmon et al., 2008). Depletion of DMPK reduces myogenin expression and prevents proper myoblast differentiation (Harmon et al., 2008). Other studies have also shown that DMPK can phosphorylate the myosin-binding subunit of myosin phosphatase (MYPT1), which inhibits myosin phosphatase (PP1c) activity (Murányi et al., 2001). DMPK also phosphorylates the SERCA2a inhibitor Phospholamban, thus regulating calcium uptake in cardiomyocytes (Kaliman et al., 2005), and Phospholemman, a regulator of Na, K-ATPase (Mounsey et al., 2000).

As for DM2, CNBP is a nucleic-acid binding protein that mainly binds single stranded DNA and RNA, and modulates the transcription of genes involved in Wnt signaling pathway (Margarit et al., 2014).

The proposed pathogenic mechanism hypothesizes that reduced DMPK and CNBP/Znf9 levels in DM1 and DM2, respectively, cause the onset of the diseases. Consistent with this, decreased amounts of DMPK transcripts and protein have been detected in DM1 patients, and this decrease inversely correlated with the CTG repeat length (Fu et al., 1993).

In this regard, DMPK knock-out mice fail to reproduce the multisystem phenotype of DM1 patients, and only develop late-onset myopathy (Jansen et al., 1996; Reddy et al., 1996; Berul et al., 1999), suggesting that haploinsufficiency of DMPK is not the primary mechanism that initiates this disease.

On the contrary, *Znf9^+/–^* mice (lacking one CNBP allele) develop a multisystem phenotype resembling DM, including muscle wasting, heart failure and cataracts (Chen et al., 2007), which suggests that CNBP insufficiency may have a role in the pathologic mechanisms of DM2 (Raheem et al., 2010). However, other studies have yielded contradictory results, indicating that aberrant CCTG expansions do not alter CNBP protein levels, and thus, CNBP deficiency would not contribute to the onset of the disease (Margolis et al., 2006).

### Cis-Alteration of Neighboring Genes

Tandem repeats produce changes in chromatin structure. Indeed, nucleosomes tend to localize in these repeat sequences, inducing chromatin condensation (Wang et al., 1994; Volle and Delaney, 2012), which could have a notable impact on the transcriptional activity of DMPK and ZNF9 flanking genes (Klesert et al., 1997; Thornton et al., 1997; Westerlaken et al., 2003). Similar to the haploinsufficiency of the repeat-containing genes in myotonic dystrophies, the altered expression of genes located in the neighborhood of the disease-causing genes could also contribute to the molecular mechanisms leading to these diseases.

In line with this, mRNA level of SIX5 (also known as DMAHP), a transcription factor coding gene located downstream of DMPK, is reduced in DM1 patients (Klesert et al., 1997; Thornton et al., 1997; Westerlaken et al., 2003). Expression of DMWD, an upstream DMPK flanking gene, also seems to be somewhat reduced in the cytoplasm of DM cells, although nuclear levels remain unchanged (Alwazzan et al., 1999; Frisch et al., 2001).

In order to ascertain the contribution of reduced levels of SIX5 to the development of DM1, a knock-out mouse model of SIX5 was created. Besides showing an increased susceptibility to developing cataracts (Klesert et al., 2000; Sarkar et al., 2000), reduced fertility in males (Sarkar et al., 2004) and altered cardiac function (Wakimoto et al., 2002), these mice do not show multisystem involvement and thus fail to recapitulate the dystrophic phenotype of DM1 patients.

### RNA-Induced Toxicity

The third pathogenic mechanism proposes that repeat expansions, once translated into RNA, exert a gain-of function toxic effect in the cells. The expanded CUG-containing transcripts form secondary structures of a hairpin shape (Michalowski et al., 1999) and sequester specific RNA-binding proteins that participate in pre-mRNA transcription and maturation, such as MBNL (Muscleblind-like) (Miller et al., 2000), thus leading to splicing defects in both DM1 and DM2 patients (Du et al., 2010; Malatesta and Meola, 2010). Double-strand RNA structures also abnormally activate the RNA-dependent protein kinase R (PKR), which in turn hyperphosphorylates CUG-BP/CELF1 protein and alters its function (Tian et al., 2000). This protein is involved in the splicing of several genes directly implicated in the multisystem phenotype of DM patients, such as cardiac Troponin (cTNT), IR and chloride channel 1 (CLCN1; Osborne et al., 2009). Overactivation of PKR also inactivates its substrate eIF2α, inhibiting the translation of specific mRNAs, such as the DNA repair factor MRG15 (Huichalaf et al., 2010).

In order to assess the contribution of foci formation to the multisystem involvement seen in DM patients, several mouse models have been created carrying variable repeat length sequences and tissue specificity. In all these models the toxicity of foci has been tested, and such models have helped to elucidate how these aggregates play a key role in the onset of the disease. For example, HSA mice, which harbor 5 (short repeat length, HSA^SR^) or 250 (long repeat length, HSA^LR^) CTG repeats in the Human Skeletal Actin gene (HSA), thus inducing a muscle-specific expression of tandem repeat-containing RNAs, have clearly established the role of RNA-protein aggregates in the development of the disease, as mice harboring long repeats developed a DM-like phenotype of muscle degeneration (Mankodi et al., 2000). RNA toxicity has been confirmed with transgenic mice harboring long triplet repeats in the DMPK gene. Such mice develop multisystem abnormalities mimicking the human DM phenotype, with predominant involvement of muscles and the CNS, although the resulting phenotype is milder than in other mouse models of the disease (Seznec et al., 2001).

Therefore, all three mechanisms may participate to some extent in the onset of a range of symptoms in DM patients, although RNA toxicity seems to contribute most notably to the multisystem degeneration seen in DM patients.

## Age-Related Genomic Events in DM

Cellular aging is accompanied by accumulating DNA damage (Moskalev et al., 2013), which includes point mutations, translocations, and double strand breaks, among others. These mutations can seriously affect the functionality of several cell types, such as the regenerative capacity of stem cells. Cells possess several mechanisms to cope with these abnormalities, either by activating DNA repair mechanisms or inducing cell death. Unfortunately, DNA damage increases with aging partially due to decreased efficiency of DNA repair systems (Gorbunova et al., 2007).

As previously mentioned, these DNA repair mechanisms participate in the expansion of tandem repeats, which cause not only DM but also other degenerative diseases such as Huntington’s Disease and Friedreich’s ataxia. Besides DNA repair mechanisms, telomere maintenance could also be implicated in muscle aging and DM.

Chromosomal ends, also known as telomeres, are composed of highly repetitive sequences that play an important role in the maintenance of chromosomal structure. Importantly, these DNA ends cannot be replicated by common DNA polymerases and thus, they continue to shorten in every cell division. Telomere shortening has been observed in most cell types during the aging of both human and mice tissues (Blasco, 2007) and has become a cellular marker of aging. However, some cell types, such as most adult stem cells, express telomerase, a specific enzyme able to replicate telomeric sequences, counteracting their shortening (Vaziri et al., 1994; Chiu et al., 1996; Morrison et al., 1996; Espejel et al., 2004; Ferrón et al., 2004; Flores et al., 2005).

Absence of telomere shortening in satellite cells of aged mice, together with the insignificant reduction of telomerase activity in aged muscle stem cells, indicates that satellite cells possess mechanisms to maintain telomeres, and thus, the age-related reduction of their regenerative potential is telomere independent. On the contrary, myogenic differentiation of these satellite cells abolishes their telomerase activity (O’Connor et al., 2009).

*In vitro* studies with human congenital DM1 samples show that despite DM1-affected satellite cells having a higher telomere shortening rate, these cells enter senescence prior to reaching critically short telomere lengths (Bigot et al., 2009; Thornell et al., 2009). In these cases, p16-dependent signaling seems to induce the senescence of satellite cells. Thus, telomere shortening, even though it is altered in DM1 patients, does not seem to play a role in the pathology of this disease. On the contrary, telomere shortening could influence the regenerative capacity of DM2-affected satellite cells, as will be discussed below Renna et al. (2014).

## Epigenetic Modifications in DMs and Aging

Epigenetic modifications encompass post-translational modifications of DNA and histones that lead to chromatin remodeling processes. Different trends of these modifications have been associated with aging, such as increased histone H4K16 acetylation, H4K20 trimethylation and H3K4 trimethylation, and decreased H3K9 methylation or H3K27 trimethylation (Fraga and Esteller, 2007; Han and Brunet, 2012; López-Otín et al., 2013). Importantly, the family of histone deacetylases known as sirtuins have been shown to possess outstanding antiaging potential (Kaeberlein, 2008; Houtkooper et al., 2012; Imai and Guarente, 2014).

Epigenetic modifications also seem to correlate with the age-dependent loss of the regenerative potential of stem cells (Liu and Rando, 2011). This also applies to muscle satellite cells, in which H3K27 trimethylation seems to increase overall in their genome, along with reduced histone biosynthesis (Liu et al., 2013).

Although epigenetic modifications in DM-affected satellite cells have not yet been studied specifically, triplet expansions seem to induce different DNA methylation patterns. In this regard, the DMPK locus shows a variably methylated sequence upstream of the triplet expansion, whereas this sequence is unmethylated in healthy controls (Ghorbani et al., 2013). Interestingly, the expanded sequence and the downstream sequence are not methylated (López Castel et al., 2011). Methylation is more frequently found in tissues of congenital and young-onset patients, although it is not limited to cells with the largest expansions or to specific tissues (López Castel et al., 2011). In an attempt to elucidate the consequences of these methylations, disruption of CTCF binding to its DNA binding sites, which flank the DM1 locus, has been postulated to explain the reduced transcription of DMPK and Six5 genes (Filippova et al., 2001; He and Todd, 2011). However, CTCF binding to DNA seems to be unaffected in a mouse model of DM1, despite the methylation and heterochromatinization found in the DM1 locus (Brouwer et al., 2013). These authors alternatively propose proliferating cell nuclear antigen (PCNA), which also binds DNA repeat sequences, as a player in the triplet-expansion mechanism.

The epigenetic modifications resulting from triplet expansions require more precise study in order to determine their contribution to the development of the disorder. The study of these modifications in muscle satellite cells would contribute to unravel the role of epigenetic modifications in the regenerative capacity of DM-affected satellite cells.

## Is Muscle Wasting in Myotonic Dystrophy a Matter of Premature Aging?

### Satellite Cells and Muscle Regeneration in DM and Aging

Satellite cells play a key role in the maintenance of muscle structure and function both in homeostasis and after acute damage. The age-related dysfunction of satellite cells has been postulated to be a key cause of sarcopenia, apart from also being implicated in other muscle wasting conditions such as muscular dystrophies (Day et al., 2010).

Satellite cells maintain skeletal muscle structure and functionality by differentiating into myogenic cells that regenerate muscle tissue (Lepper et al., 2011; Murphy et al., 2011; Sambasivan et al., 2011). In adulthood, these cells are quiescent and locate in the periphery of muscle fibers, between the basal membrane and the sarcolemma (Mauro, 1961). When muscle regeneration is required, these cells activate the cell cycle and proliferate. Most of these activated satellite cells enter the myogenic program and differentiate into myoblasts that fuse to each other or to preexisting myotubes, regenerating the damaged tissue. However, a small subpopulation of these cells repopulates the satellite cell niche (Chargé and Rudnicki, 2004; Yin et al., 2013).

Age-dependent regenerative dysfunction of skeletal muscle mainly depends on the decreased amount and functionality of satellite cells (Shefer et al., 2006; Brack and Rando, 2007; Collins et al., 2007). However, it has been widely debated if this reduced regenerative potential is due to extrinsic factors, such as age-dependent alterations of the satellite cell niche and circulating factors (Brack and Rando, 2007; Gopinath and Rando, 2008; Urciuolo et al., 2013), or due to intrinsic defects of these stem cells (Bernet et al., 2014; Sousa-Victor et al., 2014).

With regard to extrinsic factors, satellite cells are responsive to a wide variety of circulating *biomolecules*, whose concentration is constantly being modified in an age-dependent manner. Experiments involving surgical sharing of the circulating system (also known as heterochronic parabiosis) between aged and young mice have revealed that aged serum harbors factors that reduce the regenerative capacity of young satellite cells, whereas young serum is able to partially revert the myogenic-to-fibrogenic transition of aged muscle stem cells, thus reducing the muscle fibrosis seen in old muscles (Conboy et al., 2005; Brack and Rando, 2007). *In vitro* experiments performed with human satellite cells have confirmed this fact. These findings show that age-dependent systemic factors act on the regenerative potential of satellite cells (Carlson and Conboy, 2007; Carlson et al., 2009).

The satellite cell *niche*, composed of cells and extracellular matrix located in the close vicinity of satellite cells, also affects satellite cell functionality, probably through secreted factors as well as cell-to-cell interactions (Murphy et al., 2011; Naito et al., 2012). Moreover, aging alters the pattern of secreted molecules and cellular interactions, thus affecting satellite cell biology. For example, age-dependent increases in basic fibroblast growth factor (bFGF) activate satellite cells in homeostasis, inducing their differentiation, and consequently, the satellite cell pool is depleted (Chakkalakal et al., 2012). On the other hand, matrix molecules, such as extracellular fibronectin, participate in the activation of Wnt signaling in satellite cells, promoting their symmetric division (Bentzinger et al., 2013).

Apart from environmental factors, satellite cell-intrinsic factors also play a central role in the maintenance of the regenerative capacity of these cells. Recently, Sousa-Victor et al. (2014) have demonstrated that the pronounced decline in the regenerative potential of satellite cells in very old (geriatric) mice is caused by the induction of p16^Ink4a^, which drives cells from quiescence to irreversible senescence. p38 signaling also occupies a central role in the functionality of satellite cells. In this regard, activation of p38 signaling in dividing aged satellite cells induces the differentiation of both daughter cells, whereas in young animals, asymmetric localization of p38 enables satellite cells to divide asymmetrically, thus favoring the maintenance of the quiescent satellite cell pool (Bernet et al., 2014; Cosgrove et al., 2014).

Therefore, both satellite cell-extrinsic and -intrinsic factors affect the regenerative potential of muscle stem cells during aging and these factors may also play a role in DM-affected satellite cells. Indeed, it has been shown that cell-intrinsic events, such as nuclear foci accumulation, affect DM cell functionality (Malatesta, 2012), whereas the multisystem dysfunction seen in these diseases may probably alter circulating factors, impairing the regenerative potential of these cells.

Several studies have aimed to study the involvement of satellite cells in DM. In this regard, histopathologic analysis of severely affected distal muscles vs. slightly affected proximal muscles of DM1 patients has shown a two-fold increase in the number of satellite cells in severely affected muscles. Interestingly, telomere length is not altered and the number of regenerative fibers is low in both distal and proximal muscles (Thornell et al., 2009). In this regard, *in vitro* proliferative capacity of DM1 satellite cells is considerably reduced, and their entry into senescence is telomere-independent (Thornell et al., 2009). Indeed, this has been corroborated with *in vitro* cultured satellite cells from congenital DM fetuses and newborns. These satellite cells show a considerably lower proliferative rate than age-matched controls, besides having activated senescence-associated beta galactosidase, high levels of cyclin D1 and hypophosphorylated Rb. Interestingly, these cells also enter senescence prior to reaching critically short telomere lengths and express p16^Ink4a^, a Cdk4 inhibitor that induces cell cycle arrest (Bigot et al., 2009). It remains to be clarified which cellular events induce p16^Ink4a^ expression, although disturbed DNA functioning due to the formation of unusual structures, as well as increased free radicals and oxidative stress might be involved in the activation of this gene.

Besides the involvement of the proliferative capacity of DM satellite cells, the myogenic program is also abnormal in these cells. Indeed, there is evidence of the defective differentiation and maturation of DM1 myogenic progenitors *in vitro*, resulting in smaller and thinner myotubes, with a 30% lower fusion index and the lack of expression of mature myosin forms (Furling et al., 2001). This lack of fiber maturation has also been confirmed in DM1 muscle biopsies, where late myogenic differentiation markers are not fully expressed (Vattemi et al., 2005). Moreover, DM1 myoblasts seem to have impaired cell cycle withdrawal, probably due to the inability to induce the expression of p21 (Timchenko et al., 2001).

Satellite cells in DM2 show an intermediate phenotype between DM1 and healthy satellite cells. Renna et al. (2014) suggest that DM2 satellite cells also show premature entry into senescence, although later than DM1 satellite cells. Importantly, senescence seems to correlate with telomere shortening rather than the induction of p16^Ink4a^ (Renna et al., 2014). In contrast to DM1 progenitors, DM2 progenitors did not show any differentiation defects and CGUBP1 levels were also unchanged. However, DM2 cells had abnormal IR splicing (Pelletier et al., 2009). In this regard, the interaction of CUGBP1 with eIF2α and Cyclin D3-CDK4/6 seems to be crucial to achieve a correct myogenic differentiation. Reduction of these interactions in DM1 cells could explain the impaired differentiation of these cells (Salisbury et al., 2008).

This reduced differentiation and premature senescence of satellite cells resembles physiological satellite cell aging. Moreover, age-related cell-senescence features, such as cytoplasmic vacuolization, accumulation of heterochromatin and impaired pre-mRNA maturation (Malatesta and Meola, 2010) have also been found in DM cells (Malatesta et al., 2011a,b).

As previously mentioned, secreted factors also have an impact on muscle regeneration during aging, and could also play a role in DM. In this regard, congenital DM1 muscle progenitor cells with long triplet expansions seem to secrete prostaglandin, which in turn hampers myogenic differentiation, probably by lowering intracellular calcium levels (Beaulieu et al., 2012).

### Loss of Proteostasis in Aging and DM

Proteostasis encompasses cellular mechanisms that preserve the stability and functionality of its proteome in order to prevent the accumulation of damaged proteins and ensure continuous renewal of intracellular proteins. Many studies have demonstrated that protein homeostasis collapses during aging, leading to the accumulation of unfolded, aggregated and misfolded proteins, a phenomenon that causes several age-related diseases (Powers et al., 2009). Cells have various mechanisms to tackle these protein failures. The three principal proteostatic systems are the UPS, the autophagy-lysosomal system and chaperones, the efficiency of all of which decreases with aging (Calderwood et al., 2009; Tomaru et al., 2012). Consequently, old cells carry more non-enzymatic posttranslational protein modifications and accumulate more cross-linked and aggregated proteins than young cells (Soskić et al., 2008).

#### The Ubiquitin-Proteasome System

The UPS actively participates in the regulation of protein synthesis and degradation. The age-dependent decay of UPS efficiency may be the result of the reduced expression of proteasome subunits, their inadequate assembly, and/or reduced ATP availability due to mitochondrial dysfunction (Chondrogianni et al., 2014).

The activity of UPS is reduced in several aged mammals, such as humans, mice, rats and sheep. However, contrary to what could be expected, some aged tissues such as muscle, show increased expression of UPS subunits (Ferrington et al., 2005), which could be a compensatory effect for reduced constitutive proteasomal activity (Husom et al., 2004). Maintenance of proteostasis in stem cells may also play an important role in organismal aging (Vilchez et al., 2014). Indeed, proteasome activation is a conserved mechanism that regulates aging and longevity (Chondrogianni et al., 2014).

The breakdown of proteostasis has been linked to several disorders, including myotonic dystrophy. UPS is increased in skeletal and cardiac muscle of transgenic DM mice with 550 CTG repeats, triggered by the up-regulation of Fbx032/Atrogin-1 and/or Trim63/Murf1. These mice develop progressive muscle weakness between 3 and 10 months of age (Vignaud et al., 2010). Overactivation of UPS has also been confirmed in the DMSXL mouse model, which exhibits more than 1.000 CTG repeats. UPS activity was considerably increased at 4 months of age in these mice, suggesting that this proteolytic pathway could play a role in the physiopathological remodeling of muscle (Huguet et al., 2012).

Proteomic analyses have also confirmed that protein degradation is altered in DM2 myotubes (Rusconi et al., 2010). Indeed, an overall reduction in ubiquitinated proteins as well as reduced proteasome subunits have been found in DM2 myotubes (Rusconi et al., 2010). DM2 myoblasts degrade faster a variety of short-lived proteins, such as c-myc and p21, due to increased UPS activity that results from RNA CCUG repeats that bind the 20S core complex (Salisbury et al., 2009). Skeletal muscles of DM2 patients also show a deregulated *neural precursor cell expressed developmentally down-regulated protein 4* (NEDD4) ubiquitin ligase-PTEN pathway, which could contribute to the increased risk of statin-adverse reactions in patients with DM2, due to PTEN accumulation in highly atrophic muscle fibers (Screen et al., 2014).

It is worthy of note that besides UPS overactivation in DM, the 26S proteasome itself is implicated in trinucleotide expansion, thus favoring the expansion of these pathogenic repeat sequences (Concannon and Lahue, 2014).

#### Autophagy

Autophagy mediates the degradation of cellular components in order to recycle them or to obtain energy. Indeed, autophagy is activated during starvation and induces the degradation of cellular components, providing the cell with energy and thus promoting cell survival during a period of low nutrient availability. In contrast to autophagy, mTOR signaling is activated by high nutrient availability, such as insulin and amino acids, and activates cell division and protein synthesis. Importantly, age-dependent decline of autophagy disrupts cellular proteostasis.

In skeletal muscle, autophagy seems to participate in the activation of quiescent satellite cells, probably providing the additional energy required for this process (Tang et al., 2014). Thus, age-related dysfunction of autophagy could undermine satellite cell activation with aging.

*In vitro* myoblast cultures of DM1 patients show that nearly half of the myoblasts undergo abnormal differentiation. Strikingly, cells that fail to differentiate show autophagic features after 6 days in culture, with increased cellular volume and a high density of autophagic vacuoles as compared to control and DM1 differentiated cells (Beffy et al., 2010). Senescence was ruled out as an activator of autophagy in this study, as the percentage of cells expressing senescence-associated beta galactosidase was similar in DM1 cells and controls.

Autophagy may also be abnormal in DM1-affected neurons. In this regard, autophagy has been found to be activated and mTOR signaling partially inhibited in DM1-hESC-derived neurons as compared to wild type-hESC-derived neurons. Phosphorylation of the mTOR downstream components GSK3α/β and rpS6 were decreased in these cells. Importantly, reduction of mTOR signaling was p53-independent, therefore suggesting that inhibition of mTOR is not induced by cellular stress (Denis et al., 2013). In line with this, GSK3β has been found to be overactivated in muscles of the DM1 mouse model HSA^LR^ prior to the onset of muscle wasting, and GSK3β blockers improved skeletal muscle strength and reduced myotonia in this mouse model, suggesting that these inhibitors could have a beneficial effect on the treatment of DM1 by alleviating muscle wasting (Jones et al., 2012).

It has been speculated that autophagy could be a mechanism either to avoid apoptosis or to protect cells against metabolic stress. However, transfection of C2C12 myoblasts with the human DMPK-A isoform not only shows increased autophagy, but also enhances apoptosis (Oude Ophuis et al., 2009). Therefore, autophagy in DM must be thoroughly studied in order to determine the causes and the effects of its overactivation in these patients.

#### Chaperones

Chaperones exert a key function in proteostasis by folding peptides, refolding incorrectly folded proteins and unfolding damaged proteins to facilitate their degradation. Heat Shock Proteins (HSPs) constitute a subgroup of chaperones that are specifically induced by different cell stressors, such as protein damage. As expected, chaperones play a central role in protecting cells from protein damage and cell death during aging (Calderwood et al., 2009). Moreover, experiments performed in *S. cereviasiae* have shown that overexpression of specific HSPs, such as HSP104, increases protein disaggregation, reduces protein accumulation and restores UPS in aged cells (Andersson et al., 2013).

The muscle-specific HSPs HSPB3 and HSPB2, the latter also known as Myotonic dystrophy Protein Kinase Binding Protein (MKBP), seem to occupy a central role in muscle regeneration. HSBP2 specifically binds and activates MDPK, which contributes to muscle maintenance (Suzuki et al., 1998; Prabhu et al., 2012). HSBP2 and HSBP3 are induced by MyoD during myogenic differentiation, which strongly suggests that they play central roles in muscle regeneration, probably through the interaction of MKBP with DMPK (Sugiyama et al., 2000). Other HSPs, such as HSP70, may also play muscle-specific roles, as it is induced in type 1 fibers (Locke et al., 1991).

Expression of HSPB2/MKBP is specifically up-regulated in the skeletal muscle of DM1 patients, probably in order to partially compensate for the reduced amount of DMPK (Sugiyama et al., 2000). Therefore, an increase in chaperone activity would potentially benefit the maintenance of skeletal muscle functionality in both DM-affected and aged muscles.

### Mitochondrial Dysfunction

The Mitochondrial Free Radical Theory of Aging (MFRTA), which proposes that mitochondrial free radicals cause oxidative damage that gives rise to cellular aging, has been postulated as one of the main hypotheses to explain how cells age (Sanz and Stefanatos, 2008). Mitochondria play an important role in mediating and amplifying the oxidative stress that drives the aging process (Bratic and Larsson, 2013).

The largest isoform of myotonic dystrophy protein kinases, DMPK-A, supplies antioxidants and antiapoptotic signals needed for correct muscle fiber function and differentiation (van Herpen et al., 2005; Pantic et al., 2013). However, anchorage and accumulation of DMPK-A in the mitochondrial outer membrane can lead to mitochondrial fragmentation and the formation of perinuclear clusters of morphologically altered mitochondria, finally inducing the activation of autophagy (Oude Ophuis et al., 2009).

DM-patients’ muscles show a reduced expression of DMPK, mitochondrial accumulation in degenerated myofibrils and disorganization of the sarcoplasmic reticulum (Ueda et al., 1999). On the other hand, these muscles show reduced Coenzyme Q10 (CoQ10) levels, a component of the electron transport chain that participates in aerobic cellular respiration, generating energy as ATP (Siciliano et al., 2001). Blood samples have confirmed an inverse correlation between CoQ10 levels and CTG expansion length in DM patients (Tedeschi et al., 2000).

As for DM2, proteomic analyses of myotubes have detected abnormalities in two proteins involved in mitochondrial fatty acid degradation and another two proteins involved in the import, chaperonin and quality control functions of mitochondria (Rusconi et al., 2010). The elongation factors Tu and Ts, two posttranslational proteins that participate in the mitochondrial translational machinery, were also reduced in DM2 cultures. Mutations and/or reductions in these proteins are associated with muscle hypotonia and decline of motor skills (Valente et al., 2007). Therefore, mitochondrial dysfunction seems to be common to both muscular aging and DM.

### Deficiencies in Nutrient Sensing

DM patients show several metabolic defects that are also common in aged individuals, such as glucose resistance, hyperinsulinemia and in some cases, the development of diabetes mellitus. Studies performed with large DM1 samples have revealed that there are metabolic dysfunctions associated with this disease, such as primary hyperparathyroidism, calcium metabolism, thyroid insufficiency, hypogonadism, hyperprolactinemia or diabetes. Some of these dysfunctions seem to correlate with the length of the repeat expansion (Ørngreen et al., 2012).

Hyperphosphorylation of CUGBP, which is a feature of DM, leads to abnormal splicing of the IR mRNA, lacking exon 11 (Osborne et al., 2009). The IR is mainly expressed in skeletal muscle and its binding to the ligands insulin and IGF-I activates metabolic pathways implicated in muscle hypertrophy, whereas binding to IGF-II induces mitosis. This binding affinity is isoform-dependent; the form that lacks exon 11 (immature form), is mainly expressed in embryonic tissues and shows high affinity towards IGF-II, compared to insulin and IGF-I. Due to the abnormal splicing, DM skeletal muscles are characterized by a predominant expression of the immature isoform, which leads to insulin insensitivity (Savkur et al., 2001, 2004). This splicing defect seems to be independent of muscle fiber type, as both fiber types show a reduced expression of the adult IR isoform (Santoro et al., 2013), despite DM muscle wasting being fiber-type dependent (Vihola et al., 2003; Pisani et al., 2008).

These splicing abnormalities have been observed in muscle tissue and myotube cultures of both DM1 and DM2 patients prior to the development of muscle histopathology, which indicates that DM1 and DM2 share common pathogenic mechanisms and that these splicing abnormalities appear before myofiber degeneration (Santoro et al., 2013).

Insulin secretion has also been found to be abnormal in DM patients, probably due to loss of calcium homeostasis that regulates insulin secretion by pancreatic beta-cells (Savkur et al., 2001).

Thus, deficiencies in nutrient sensing are shared by both physiological aging and DM patients, which leads to the use of common therapeutic approaches to treat both conditions.

## Concluding Remarks

Myotonic dystrophies represent a new paradigm of how a genetically determined disease initiates a cascade of events that lead to a wide variety of symptoms (myotonia, cataracts, heart dysfunction, baldness, etc.) that resemble the multisystem involvement induced by aging.

Different pathogenic mechanisms exist to explain how these tandem expansions in the genome of affected patients lead to the DM phenotype, although it has not yet been clearly defined to what degree each mechanism contributes to the development of the disease.

**Figure 4:**
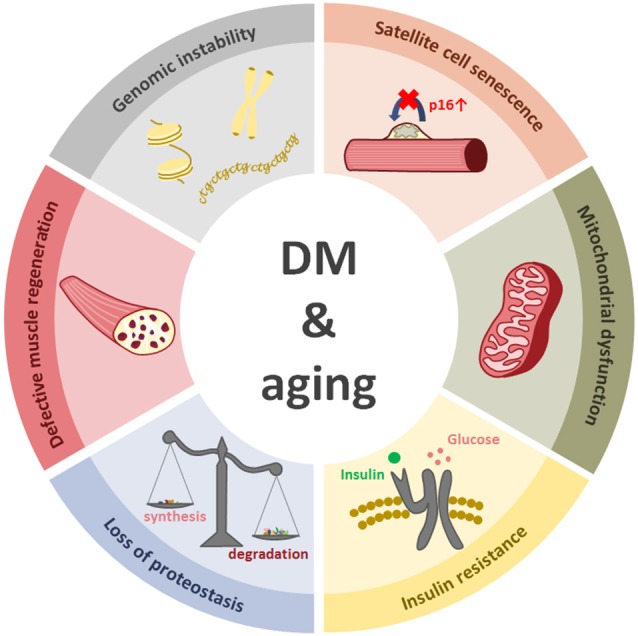
**The figure represents six cellular events that happen in both aging and myotonic dystrophy**.

An important feature of myotonic dystrophies and aging resides in their progressive nature. The described molecular events, such as genomic instability, alteration of autophagy or mitochondrial dysfunction, among others, induce cell damage that continues to accumulate throughout life (Figure 4).

Satellite cells have been hypothesized to be the main contributors to muscle regeneration. Numerous studies have established that both cell-intrinsic and environmental factors induce the age-related decline of their regenerative capacity in muscle aging and sarcopenia. However, it is still unclear how these cells behave in different muscle dystrophies and if their loss of regenerative potential is crucial in muscle wasting seen in affected patients.

The molecular pathologic mechanisms of DM, as well as the aging-related events reviewed above, strongly suggest that satellite cell dysfunction could be a major contributor to the development of muscle wasting in these patients and thus, that these cells could become potential targets for the treatment of both age-related and DM-induced muscle dysfunction. It is worth highlighting that skeletal muscle fibers also present proteostatic and mitochondrial abnormalities that resemble general aging processes. Thus, DM patients and animal models can be considered* bona fide* models of aging, and this should be kept in mind when designing treatments to treat both myotonic dystrophy patients and aging-derived disorders.

## Author Contributions

All authors have contributed to the design, data collection and writing of the manuscript. Authors have critically revised the work and they have approved the submitted version of the review.

## Conflict of Interest Statement

The authors declare that the research was conducted in the absence of any commercial or financial relationships that could be construed as a potential conflict of interest.
